# Comprehensive Appraisal of Graphene–Oxide Ratio in Porous Biopolymer Hybrids Targeting Bone-Tissue Regeneration

**DOI:** 10.3390/nano10081444

**Published:** 2020-07-24

**Authors:** George Mihail Vlasceanu, Aida Șelaru, Sorina Dinescu, Cornel Balta, Hildegard Herman, Sami Gharbia, Anca Hermenean, Mariana Ionita, Marieta Costache

**Affiliations:** 1 Faculty of Medical Engineering, University Politehnica of Bucharest, Gh. Polizu 1-7, 011061 Bucharest, Romania; vlasceanu.georgemihail@yahoo.ro; 2 Advanced Polymer Materials Group, University Politehnica of Bucharest, Gh. Polizu 1-7, 011061 Bucharest, Romania; 3 Department of Biochemistry and Molecular Biology, University of Bucharest, Spl. Independentei 91-95, 050095 Bucharest, Romania; aida.selaru@bio.unibuc.ro (A.Ș.); sorina.dinescu@bio.unibuc.ro (S.D.); samithgh2@hotmail.com (S.G.); anca.hermenean@gmail.com (A.H.); marietacostache@gmail.com (M.C.); 4 Department of Immunology, National Institute for Research and Development in Biomedical Pathology and Biomedical Sciences “Victor Babes”, Spl. Independentei 99-101, 050096 Bucharest, Romania; 5 Research Institute of the University of Bucharest, 050095 Bucharest, Romania; 6 Aurel Ardelean Institute of Life Sciences, Vasile Godis Western University of Arad, Rebreanu 86, 310414 Arad, Romania; baltacornel@gmail.com (C.B.); hildegard.i.herman@gmail.com (H.H.)

**Keywords:** polymer composite, polymer blend, graphene oxide, chitosan, fish gelatin, bone tissue engineering, biocompatibility, microcomputer tomography

## Abstract

The bone-tissue engineering (BTE) field is continuously growing due to a major need for bone substitutes in cases of serious traumas, when the bone tissue has reduced capacity for self-regeneration. So far, graphene oxide (GO)-reinforced natural materials provide satisfactory results for BTE, for both in vitro and in vivo conditions. In this study, we aimed to evaluate the biocompatibility of a new biocomposite consisting of chitosan and fish gelatin crosslinked with genipin and loaded with various concentrations of GO (0.5, 1, 2, 3 wt.%) for prospective BTE applications. Scaffold characterizations revealed a constant swelling degree and good resistance to enzyme degradation. The composites presented a porous structure with pores of similar size, thus mimicking the bone structure. In vitro biocompatibility assays demonstrated an overall beneficial interaction between preosteoblasts, and these particular composites, particularly with 0.5 wt.% GO, reinforced composition. Next, the materials were implanted subcutaneously in 6-week old CD1 mice for in vivo evaluation of biocompatibility and inflammatory activity. Immunohistochemical staining revealed maximal cell infiltration and minimal inflammatory reaction for fish gelatin/chitosan/genipin with 0.5 wt.% GO scaffold, thus demonstrating the best biocompatibility for this particular composition, confirming the in vitro results. This study revealed the potential use of fish gelatin/chitosan GO composites for further implementation in the BTE field.

## 1. Introduction

Traumatizing skeletal anatomy—the hard, mineralized, interconnected tissue which facilitates locomotion, with crucial functions in the body (protection of vital organs as well as calcium and phosphorus levels regulation) [1]—severely alters the patient’s quality of life. Bone tissue has the ability to self-regenerate after minor injuries or small fractures. From the moment of the trauma, the process of healing consists of five stages and lasts from two months to two years, depending on the seriousness of the injury [2]. Yet, if the lesions in the bone are major, the tissue will not have the capacity to heal on its own. These kind of surgical interventions are very meticulous, and the donor can suffer from morbidity at the sampling site. Therefore, the field of bone-tissue engineering (BTE) seems to hold great promise in reducing patient discomfort and surgical risks, as well as minimizing costs. However, BTE is epitomized by three commanding features: (1) a scaffold that mimics the structure of bone extracellular matrix (bECM); (2) a cell source that can follow the bone lineage, and (3) growth factors to support cell growth and development [3], which often prove to be delicate in addressing altogether.

Lately, tremendous efforts have been made in generating tridimensional (3D) biomaterials based on natural and synthetic compounds for BTE. These substitutes need to have specific proprieties, namely, to be biocompatible, biodegradable, and fashionable into designs that mimic native bone morphology in order to provide a familiar environment for tissue regeneration. It is imperative for an implanted scaffold to harmoniously interact with cells, not to generate significant inflammatory response, and—upon completing cell integration—to degrade without releasing toxic species [4].

In the research and development field, numerous polymer and composite blends have been reported as capable of enacting physiological signals with regenerative outcomes. However, most of these studies are indefinite and exhaustive conclusions are yet to arise. Blending has become a common approach in designing structures with appropriated features on the principle of complementarity. In this context, it is preferred to opt for natural polymer formulations. Among them, gelatin (G) and chitosan (Cs) emerged as robust materials for bone regeneration support due to the affinity shown by various cell types used in BTE [5]. Chitosan has been safely used to fabricate tridimensional scaffolds with biomineralization promoting abilities that elude a post-implantation inflammatory response [6]. Gelatins, structurally derived from collagen, naturally found in bECM too, can support osseous regeneration due to the affinity cells manifest towards its reactive side chain radicals and arginine–glycine–aspartate (RGD) sequence in particular [7]. Aquatic gelatins, like ones derived from cold-water fish, provide a series of advantages over mammalian strains, which could be less microbiologically safe and become vehicles for viral outbreaks in particular and are conflicting with the spiritual conviction of billions (kosher, halal, or ahimsa) and are less versatile with respect to solubility and viscosity in variable environmental conditions [8]. Both macromolecular compounds have been studied for decades, apart and in combination. Genipin (Gp), a naturally occurring compound, was preferred as a substitute to typical crosslinking agents for its lower cytotoxicity and potency to stabilize primary amine group molecules [9]. In tissue engineering, the appealing route that involves Gp crosslinking of Cs and mammalian G networks was previously described as a mean to ensure the support for various cell cultures, including neuroblastoma cells [10], HepG2 [11], or fibroblasts [12] with short in-depth reflection on the hybrid’s perspectives on other types of tissue reconstructions. To the best of our knowledge, only one group [13] has taken up the assessment of GCs uncrosslinked networks loaded with graphene oxide (GO) for BTE prospects.

GO is a monolayered carbonaceous nanostructure consisting of a single atomic sheet of sp^2^ hybridized atoms promising for substrate fabrication in the field of BTE [14]. GO associates with proteins via electrostatic and hydrophobic interactions, which probably provide structural bone-oriented matrices with osteogenic differentiation cues for progenitor lines [15,16]. At the same time, GO induced cytotoxicity has been reported [17], with little understanding on the short- and long-time outcomes, suggesting that GO use comes with risks that need better exploration.

In restorative medicine studies, due to the complex molecular structuration and the patterning particularities of the living matter, hybrids are the closest commodities to address biomimicry, which is pivotal in robust scaffold fabrication. In this context, the aim of this study was to undertake the fabrication and characterization of 3D freeze-dried scaffolds consisting of chitosan/fish gelatin/genipin enriched with various ratios of GO and to assess its biocompatibility in vitro with murine preosteoblasts, as well as in vivo on CD1 mouse models with respect to GO proportions. The two controls and composite 3D architectures hereby synthesized and described next will be referred to as: GCs for gelatin-chitosan, GCsGp for genipin-crosslinked gelatin-chitosan, GCsGp/GO (0.5/1/2/3) for genipin-crosslinked chitosan-gelatin with 0.5, 1, 2, and 3 wt.% GO supplementation. Our group have previously undertaken a pilot survey of these compositions from the standpoint of material structuration and thermomechanical alterations to determine whether GO is able to tailor the properties of the blend in a constructive manner [18]. With this, however, we round up the investigation of these bio-based hybrid composites by steering the focus towards the implications of GO additivation in porogenesis, morphology and cellular response, and internal medium behavior.

## 2. Materials and Methods

### 2.1. Materials

Graphene-oxide powder, Chitosan (medium molecular weight), cold water fish Gelatin, HPLC-grade Genipin (≥98%), and Acetic acid (≥99.7%) were procured from Sigma/Merck and used without additional purification. Double distilled water was used throughout the experiment. Collagenase (lyophilized powder from *Clostridium histolyticum*, ≥125 CDU/mg solid), Sodium azide (≥99.5%), Calcium chloride (≥97%), Tris-HCl, and Ethylenediaminetetraacetic acid (≥99%) were purchased from Sigma/Merckand and used without additional purification. Phosphate Buffer Saline (powder, pH 7.4) was used to prepare the aqueous (double-distilled water) solution for sample hydration prior to the degradation onset.

### 2.2. Material Synthesis

GCsGp samples with GO concentrations of 0.5, 1, 2 and 3 wt.% were prepared and crosslinked by 0.5 wt.% Gp solution. 1 wt.% Cs and 5 wt.% G were used to a dry polymer mass ratio of 1:1, while GO and Gp concentrations were related to the total polymer mass. For each individual sample, a volume of 50 mL of G-CS mixture was chosen. Synthesis was initiated with the preparation of 1 wt.% Cs solution by dissolving Cs powder in a diluted acetic acid solution of 1 wt.% at 50 °C. Separately, the corresponding GO amount for each sample was added to 8.33 mL of distilled water and dispersed by sonication for 1 h. Thereafter, G was dissolved in the GO–water dispersion by thoroughly stirring at 50 °C for 1 h. A volume of 41.66 mL CS was added within the G solutions and mixed at room temperature for another 30 min. The GCs/GO composite solutions were eventually crosslinked by adding 0.42 mL of Gp solution (1% in water), casted in Petri dishes, left undisturbed for 12 h, frozen at −80 °C, and lyophilized for 72 at −55 centigrade and 0.28 mbar.

The GO exfoliation was carried out using a VCX 750 ultrasonic device from Sonics & Materials, Inc. (53 Church Hill Road, Newton, CT 06470-1614 USA) provided with a Ti-6Al-4V probe tip and a 750 W processor operating at 20 kHz. The amplitude of the probe tip vibrations was set at 70% throughout the 1 h GO exfoliation procedure.

### 2.3. Material Characterization

#### 2.3.1. Swelling Degree Assessment

The swelling behavior of the porous scaffolds was performed by rehydration in phosphate buffer saline (PBS). In brief, samples from each material combination were weighed to determine the dry weight (Md) and then immersed in freshly prepared PBS pH 7.4 at 37 °C, in quadruplicate. The wet weight (Mw) was measured after 10′, 20′, 40′, 1 h, 2 h, 4 h, 8 h, 12 h, and 24 h post immersion, to assess the profile of water uptake and to estimate the PBS equilibrium content. The measured values of Md and Mw were used to ascertain the swelling degree (%SD) after rehydration by applying Equation (1). The resulted data was the support for the enzyme degradation.

%SD = 100 × (Mw − Md)/(Md)(1)

#### 2.3.2. Gel Fraction Estimation

The enzymatic degradation is based on a previously described method [19] carried out under static conditions. Briefly, the in vitro enzyme degradation was carried out by incubating the equal specimens in 0.5 mL of Tris-HCl (0.1 M, pH 7.4) buffer solution with 0.005% (*w/v*) NaN_3_ and 5 mM CaCl_2_ for 1 h, followed by the addition of 0.5 mL of collagenase solution (200 µg/mL). Since collagenase type II activity is temperature dependent, the experiment was conducted at physiological temperature (37 °C) in quadruplicates for each composite formulation and controls and then averaged. Sample degradation was stopped by collagenase inactivation after 10′, 20′, 40′, 1 h, 2 h, 4 h, 8 h, 12 h, 24 h, 72 h, 7 days, 30 days, 60 days, and 90 days by the addition of 0.1 mL of 0.25 M ice-cold EDTA solution and accelerated cooling in an ice bath. Upon collagenase quenching, samples were rinsed three times with ice-cold Tris-HCl and ice-cold double-distilled water. Gel fraction (GF) was determined after sample drying in the air at 37 °C by employing the Equation (2) where Wd,t is the weight of the dried sample at time, t, of degradation, and W0 is the initial weight of the sample.

%GF = (Wd,t/W0) × 100(2)

#### 2.3.3. Scanning Electron Microscopy (SEM)

The morpho-structural investigations of gold-sputtered GCs, GCsGp, and GCsGp/GO (0.5/1/2/3) porous scaffolds were performed by using the Quanta Inspect F SEM device equipped with a field emission gun (FEG) (Fei Company, Hillsboro, OR, USA) with 1.2 nm resolution.

#### 2.3.4. Microcomputer Tomography (µCT)

For the microcomputer tomography analysis, Bruker µCT 1272 high-resolution equipment was used. The scanning was carried out without filter, source voltage was set at 45 kV, current intensity at 200 µA, while the exposure per frame was set at 550 ms. The scanning was performed while samples rotated with 180°, with a rotation step of 0.15°. For each individual slice, the image was the result of averaging 6 frame acquisitions. For the 6 samples, the image pixel size was fixed at the value of 5 µm.

Tomograms were reconstructed from the raw data in Bruker NRecon 1.7.1.6 software (Kontich, Belgium). Generally, beam hardening correction was set to 25, ring artefact reduction to 17 and smoothing to 1. Reconstructed tomograms were rendered in CTVox (Bruker), while sample analysis was performed in CTAn 1.17.7.2 software (Bruker, Kontich, Belgium). For each composite, 5 cylindrical volume-of-interest (VOI) datasets were extracted. The VOIs were constrained in terms of diameter (4 mm) and height (500 slices). For the 3D analysis in CTAn, the VOIs were subjected to an image-processing task list consisting of thresholding (to singularly separate the specimen walls from its pores), despeckling (for the removal of remnant scanning artefacts), and 3D analysis (for the numeral quantification of specific surface (Sp.S), total porosity (T.Po), structure separation (St.Sp), and structure thickness (St.Th)). No other image processing technique was applied. Numerical results in Table 1 represent the mean values of T.Po, St.Th, and Sp.S recorded for the 5 VOIs of each composite scaffold with standard deviation.

#### 2.3.5. GCSGp/GO Bioconstructs’ Achievement

3D scaffolds were exposed to UV light for sterilization and then incubated with a complete culture medium overnight. MC 3T3-E1 preosteoblast cell line (ATCC CRL 2593TM) was grown in Dulbecco’s Modified Eagle Medium (DMEM) supplemented with 1% antibiotic (Sigma/Merck, Steinheim, Germany) and 10% fetal bovine serum (FBS, Life Technologies, Foster City, CA, USA). Cells were seeded on the materials surface at a density of 2 × 10^5^ cells/cm^2^, resulting in biosystems. These were further maintained in standard culture conditions (37 °C in humidity and 5% CO_2_) for 7 days, during which the materials were assessed for biocompatibility.

#### 2.3.6. In Vitro Biocompatibility Assessment

Biocompatibility studies were performed after 3 and 7 days of culture in standard conditions and included quantitative assays, such as methylthiazolyldiphenyl tetrazolium bromide (MTT) and Lactic Dehydrogenase (LDH) tests, correlated with a qualitative visual analysis and a Live/Dead assay, in order to simultaneously visualize the amount of live (green-labeled) cells and dead (red-labeled) cells were in the scaffolds.

The metabolic activity of living cells was assessed using methylthiazolyldiphenyl tetrazolium bromide (Sigma/Merck, Steinheim, Germany). The solution was prepared at the recommended concentration of 1 mg/mL in culture media lacking FBS. After 4 h of incubation with MTT solution, the resulted formazan crystals were dissolved using isopropanol to a final violet solution, which was measured by spectrophotometry at 550 nm using FlexStation3 (Molecular Devices, San Jose, CA, USA).

Cell membrane integrity was assessed using the “In Vitro Toxicology Assay kit, Lactic Dehydrogenase Based” TOX7 kit (Sigma/Merck, Steinheim, Germany). The solutions were prepared following the manufacturer instructions and the final product was measured by spectrophotometry at 490 nm, using FlexStation3 (Molecular Devices, San Jose, CA, USA). The positive control for this assay was represented by a 3T3-E1/GCs biosystem, which was treated with 2% Triton-X100 (Sigma/Merck, Steinheim, Germany) in order to induce 100% of cell cytotoxicity. The final results were carried out in percentages based on their absorbance, comparing them to the positive control (100% cytotoxicity).

To qualitatively assay the living and dead cells, Live/Dead kit “LIVE/DEAD™ Viability/Cytotoxicity Kit, for mammalian cells” (Thermo Fisher Scientific, Foster City, CA, USA) staining was performed. The staining solution was prepared following manufacturer instructions and used as incubation media of the bioconstructs for 40 min in the dark, at room temperature. The sample examination was performed using a laser-scanning confocal microscope (Carl Zeiss LSM 710 system, Zeiss, Germany) and images were analyzed using corresponding Zeiss Zen 2010 software (LSM710 software ZEN2010, Zeiss, Jena, Germany).

#### 2.3.7. In Vivo Biocompatibility Assessment

CD1 mice (6 weeks old, weight 20–25 g) from the Animal Facility of Vasile Goldis Western University of Arad were used. Animal handling was carried out in accordance with the European Union Directive 2010/63/EU, and all experimental procedures were approved by the National Sanitary Veterinary and Food Safety Authority (ANSVSA).

Animals were housed in groups of three in IVC ventilated cages, with ad libitum access to water and food and a light/dark cycle of 12/12 h. Surgical procedures were done under aseptic conditions, keeping mice under anesthesia by intraperitoneal (i.p.) administration of 100 mg/kg ketamine hydrochloride and 10 mg/kg b.w. xylazine hydrochloride.

Scaffolds were implanted into a subcutaneous pocket in the dorsum of the animals. The implanted mice were randomly assigned to six groups (*n* = 10/group): GCS, GCSGp, GCSGp/GO 0.5%, GCSGp/GO 1%, GCSGp/GO 2%, and GCSGp/GO 3%. All the mice were euthanatized by anesthetic overdose 4 weeks after surgery. The scaffolds were explanted and collected for analysis.

For the histopathological study, explant samples were fixed in 4% paraformaldehyde solution in PBS, embedded in paraffin and stained with Gomori’s trichrome kit (Leica Biosystems, 38016SS1, Nussloch, Germany). Microscopic sections were analyzed with an Olympus BX43 microscope.

Paraffin-embedded sections of 5 μm thickness, previously deparaffinized and rehydrated using a standard technique, were exposed to a rabbit polyclonal anti-CD80 antibody diluted 1:40 (Abcam) and an anti-mannose receptor (CD206) antibody diluted 1:100 (Abcam), as primary antibodies. Immunoreactions were visualized employing a Novocastra Peroxidase/DAB kit (Leica Biosystems, Nussloch, Germany), according to the manufacturer instructions. Stained slides were analyzed with an Olympus BX43 microscope.

#### 2.3.8. Statistical Analysis

All biocompatibility experiments were performed in triplicate (*n* = 3), and the results were expressed as a ± standard deviation (SD) using Graph Pad Prism Software 3.0 (Graph Pad Software Inc., San Diego, CA, USA). Statistical relevance was assessed using the same software, the one-way ANOVA method, and the Bonferroni post-test, and significant statistical differences were considered for *p* < 0.05.

## 3. Results

### 3.1. Swelling Degree Assessment

Graphene-biopolymer composite networks swell and soften in simulated physiological environments, such as, PBS without dissolving. To assess both shape, stability, and swelling capacity of the 6 formulations and predicate their behavior post implantation, preweighted specimens were immersed in PBS solution (pH 7.4, 37 °C). At different time intervals, the specimens were weighted after the excess buffer was purged from the surface. The scheme was repeated until weight stabilization.

**Figure 1:**
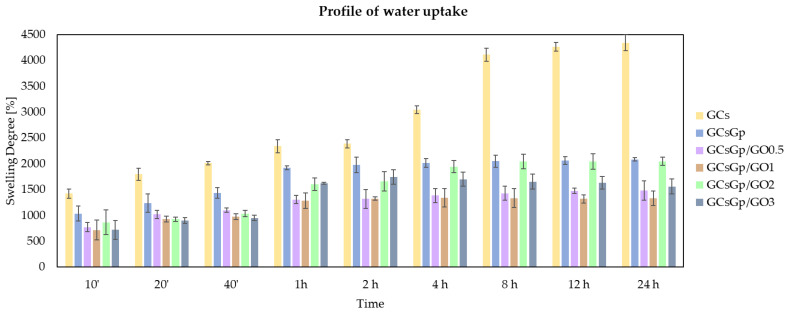
Swelling behavior of GCs, GCsGp, GCsGp/GO 0.5%, GCsGp/GO 1%, GCsGp/GO 2%, and GCsGp/GO 3%.

A time-dependent water uptake survey is charted in Figure 1 for all compositions, since the ability to rehydrate post dehydration is exhaustive regardless of GO supplementation. At the end of PBS incubation, the obtained composite constructs preserved their original shape, the only fragmentation that occurred was in the case of GCs specimens, proving that the networks are stable due to the efficient crosslinking process. The pristine and GO enhanced compositions exhibited different rates of swelling before reaching the maximum SD. The initial rapid swelling indicated that a state of equilibrium would be reached in 1 h, however GCs and some GO-rich gels regulated their water uptake profile within 12 h and remained constant thereafter. The measured swelling degrees were in the range of 14–44 g PBS solution/g solid gel whereby uncrosslinked GCs was the highest (probably due to the highest amount of amine and imine functionalities available for protonation [13]) and GCsGp/GO 1 in the lowest. Water uptake was highly influenced by the Gp crosslinking as the average hydration degree of GCsGp was half (21.5 g PBS solution/g solid content) of the GCs’. 

### 3.2. Gel Fraction Estimation

The in vitro stability of the six compositions was assessed in simulated biological fluid enriched with type II collagenase (COLase II). In vitro assessment of COLase II activity should be discussed, considering also the physical conditions of the experiment. The incubation was static and did not involve mechanical loads or spread restraints common in clinical live models. As a result, the degree of sample expansion is not conditioned in space; COLase II percolation is favored at microscale, and molecular reach and its thrust is maximized in vitro. A complex cellular environment also has the benefit of favoring a prolonged and regulated degradation: after the artificial cell support is populated, the extracellular matrix enveloping the implant contributes to balancing the opposite processes of tissue regeneration and scaffold degradation.

By visually inspecting the process, it was perceived that complete degradation of the materials did not occur before 72 h and only for GCs. However, the survey made apparent reduced cohesiveness in all freeze-dried specimens after 4–12 h of incubation. COLase II exposure of more than 3 days induced dramatic changes in the morphological integrity of the specimens that were due to both swelling and an enzyme attack. Nonetheless, the maneuverability of the GO composites and chemically crosslinked reference, though demanding, was possible without altering their unitary integrity 3 months after COLase II quenching.

**Figure 2:**
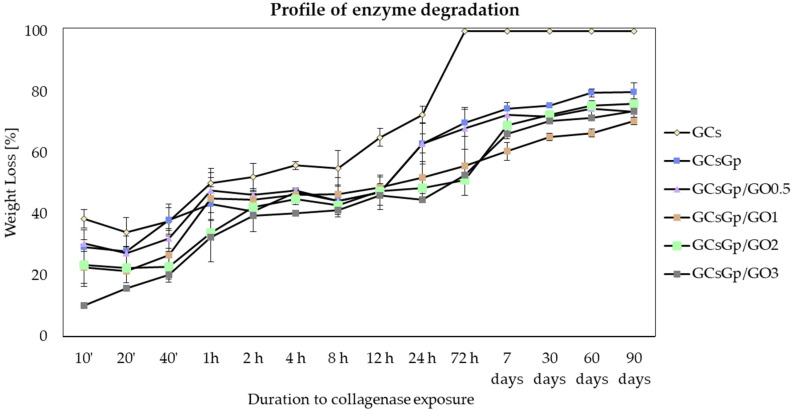
Degradation profile of GCs, GCsGp, GCsGp/GO 0.5%, GCsGp/GO 1%, GCsGp/GO 2%, and GCsGp/GO 3% in the presence of type II collagenase.

GCs’ reference is the most susceptible for degradation, followed by crosslinked control and 0.5% GO additivated composition in alike rates. The initial contact of GCs with COLase II determined a weight loss of about 55% in less than 8 h that increased to 72% in 24 h; enhanced resistance was outlined for Gp’s stabilized reference. With respect to the crosslinked batch, the behavior is not surprising considering that crosslinking enables the fabrication of implantable biodevices with superior output in tissue restauration; still, elevated crosslinking should be paid attention to since it could generate upraised foreign body response. Low amounts of GO (0.5 wt.%) slightly induced behavioral changes onto GCsGp formulation, however the increase of GO content in the composite networks supported the intrinsic resistance of the materials to COLase II. Chemically crosslinked materials with 1, 2, and 3 wt.% GO better withheld COLase II degradation during 24 h exposure (44–52% weight loss), per the depiction in Figure 2.

### 3.3. Morpho-Structural Investigations

**Figure 3:**
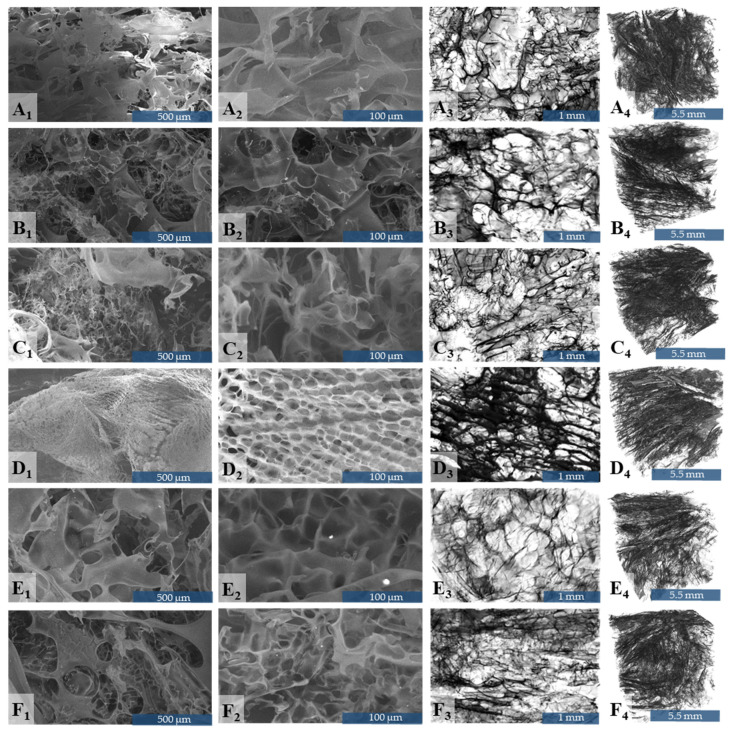
Morphological characterization of GCs (**A**), GCsGp (**B**), GCsGp/GO 0.5% (**C**), GCsGp/GO 1% (**D**) GCsGp/GO 2% (**E**), and GCsGp/GO 3% (**F**) through SEM (1–2) and µCT (3–4).

Morpho-structural features of biopolymer-GO dry-state composite porous scaffolds were investigated by scanning electron microscopy and microcomputer tomography. In Figure 3, the internal architecture of the scaffolds is illustrated. In brief, all freeze-dried composites exhibited highly interconnected pore networks (Figure 3A–F). Through visual inspection, the pore size distribution seems broad. Upon GO load, at higher magnifications (Figure 3C2–F2), the biopolymer matrix develops a propensity to organize in an ordered fashion. Pores of similar size significantly oriented in parallel patterns that resemble bone structure. The occurrence of this periodic architecture within the porous scaffold was, however, intermittent. Thin pore walls were noticed in all compositions. Even though the wall morphology was sinuous, topographically their surface was smooth, with little rough protrusions and rather curved edges.

**Figure 4:**
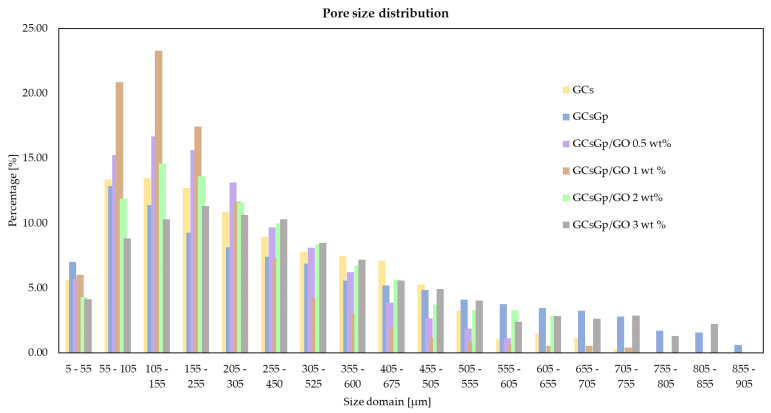
Rendering of the averaged pore size in GCs, GCsGp, GCsGp/GO 0.5%, GCsGp/GO 1%, GCsGp/GO 2%, and GCsGp/GO 3% specimens measured in CTAn software from the 3D tomograms.

In Figure 3, CTVox 3D displays of GCsGp/GO volumes are rendered with reciprocal 2D thin slice illustrating materials dominant and bone-like morphology. Scale was fixed for all six formulations. The architecture of the freeze-dried specimens is porous, almost entirely interconnected, highly anisotropic, featuring random pore orientation. Referring to the total pore volume of the scanned object, closed porosity extended to less than 0.1%. Table 1 summarizes total porosity, average wall thickness, and specific surface. Specific surface was asserted as the ratio between all walls’ surface and object (walls and pores) volume, hence it is heavily influenced by the magnitude of porosity, while Figure 4 depicts the weighted-average pore-size distribution within the six compositions.

Pore size distribution within the GCs batch is illustrated in Figure 4. The measurements resulted from 3D tomograms processing in CTAn (Bruker company software) by means of thresholding (clear separation of solid matter from aerial environment), despeckling (scanning artefacts reduction to minimal values), and 3D analysis (distinct quantifications of the number of three dimensional pixels associated to the physical object and pores). 3D analysis is able to provide metric values for various morphological features of the scanned object by converting the size of the 3D pixel (called voxel) to standard units according to the scanning resolution. All objects were scanned at the same resolution of 5 µm, which entails that a pixel of the 2D-acquired images covers 5 µm from the sample (and a voxel a volume of 125 µm^3^). For clarity, values were assembled in 50 µm domains and plotted in a histogram.

All six distributions followed a left-skewed Gaussian arrangement. The dimensional array was more or less broad considering the composite content and GO share. There was no proportionality between GO content and the size–value range, however a bell-flattening trend arose. In particular, with GO content increase, more dimensionally ordered pore domains occur. Porogenesis in GCsGp/GO composites underwent a templating refinement meaning that the pore dimensional range evened and the ratio of larger pores marginally increased. The presence of larger pores in implantable devices is favorable for cell migration mitigated also by a smoother flow of nutrients and bioactive factors.

### 3.4. Preosteoblast Viability Evaluation in Contact with GCS/GO Scaffolds

**Figure 5:**
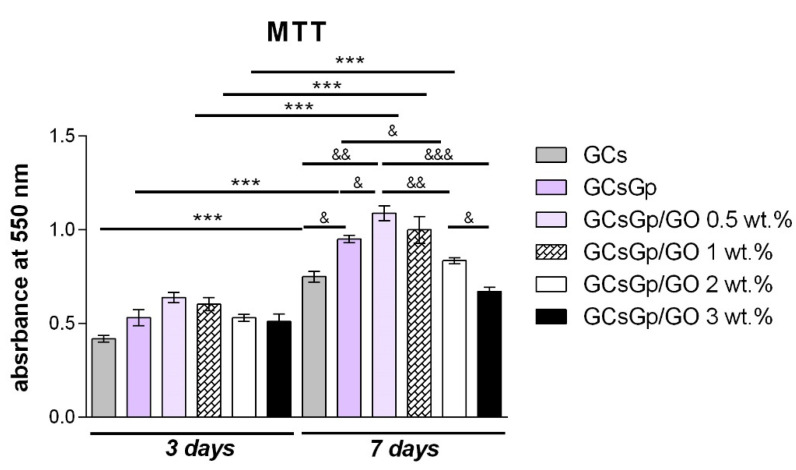
Murine preosteoblasts viability and proliferation profile as resulted from quantitative evaluation by MTT assay after 3 and 7 days of in vitro cell culture. Statistical significance: &—*p* < 0.05; &&—*p* < 0.01, *** and &&&—*p* < 0.001 (“*” was used to compare the viability and proliferation profile of cells on the same material but at different time points during the experiment, and “&” was used to compare the cell viability on different materials during the same time).

Cell viability and proliferation assessment via MTT assay (Figure 5) indicated an overall good interaction between murine preosteoblasts and GCsGp/GO scaffolds after 3 days. Even though some differences in cell behavior are visible among the composites, no significant differences were registered the first 3 days of culture. On the other hand, the MTT profile changed after 7 days of culture, firstly indicating a significantly (*p* < 0.05) higher cell viability in contact with GCsGp than in contact with GCS control. Moreover, it can be observed how the amount of living cells was significantly (*p* < 0.01) increased on the composite enriched with 0.5 wt.% GO as compared to neat GCs control. Between GCsGp/GO 2 and 3 wt.% and GCs, there were no significant cell viability differences found. When comparing GO-enriched scaffolds with GCs control, cell metabolic activity was found to be significantly (*p* < 0.05) increased starting with the addition of 0.5 wt.% GO. In contrast, the addition of 2 wt.% GO led to a decrease in cell viability (*p* < 0.05), suggesting that higher concentrations of GO could negatively affect cell metabolism and viability. Concerning the GO-enriched scaffolds, it can be observed that between GCsGp/GO 0.5 wt.% and GCsGp/GO 1 wt.% no significant change in cell viability was found. Contrarily, GCsGp/GO 2 wt.% and GCSGp/GO 3 wt.% exposed a significantly (*p* < 0.01 and *p* < 0.001, respectively) lower viability when compared to GCsGp/GO 0.5 wt.%. MTT assay results also indicated that murine preosteoblasts maintained their viability throughout one week of culture and that they started to proliferate from 3 to 7 days. Interestingly, all tested composites and controls, besides GCsGp/GO 3 wt.%, presented a significant (*p* < 0.001) proliferation rate from 3 to 7 days post seeding.

### 3.5. GCS/GO Cytotoxicity Assessment

**Figure 6:**
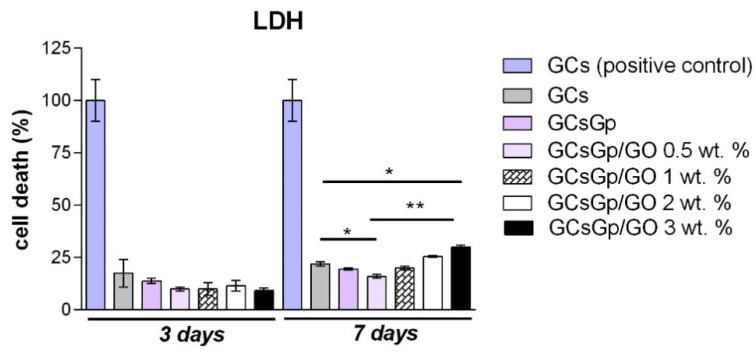
GCs and GCsGp/GO scaffolds’ cytotoxicity evaluation by LDH assay during 7 days of in vitro cell culture. Statistical significance: *—*p* < 0.05; **—*p* < 0.01.

According to the LDH assay (Figure 6), it was noticed that overall all GCsGp materials containing GO exhibited a certain level of cytotoxicity. When compared to the positive control 3 days post seeding, comparable levels of LDH were found on GCs, as well as on scaffolds enriched with GO. Between the two tested controls, GCs and GCsGp, no significant differences were found. GO-enriched composites exhibited low levels of cytotoxicity, with no significant differences between them. At 7 days of culture, higher levels were found, depending on the GO concentration. Similar levels of cytotoxicity were found for controls and GCsGp/GO 1 and 2 wt.%, while the lowest value was registered for GCsGp/GO 0.5 wt.% (*p* < 0.05 as compared to GCS control). Moreover, significantly (*p* < 0.05) high levels of toxicity were found for GCsGp/GO 3 wt.%, as compared to GCs control, showing that high levels of GO are able to exert different cell responses and release higher LDH levels. Overall, these materials exhibited a cytotoxicity of 25–30% as compared to the 100% toxic positive control, suggesting a significant difference, which demonstrates that these materials are not toxic and biocompatible with murine preosteoblast.

### 3.6. Live and Dead Cells Ratio as Response to GCsGp/GO Materials

**Figure 7:**
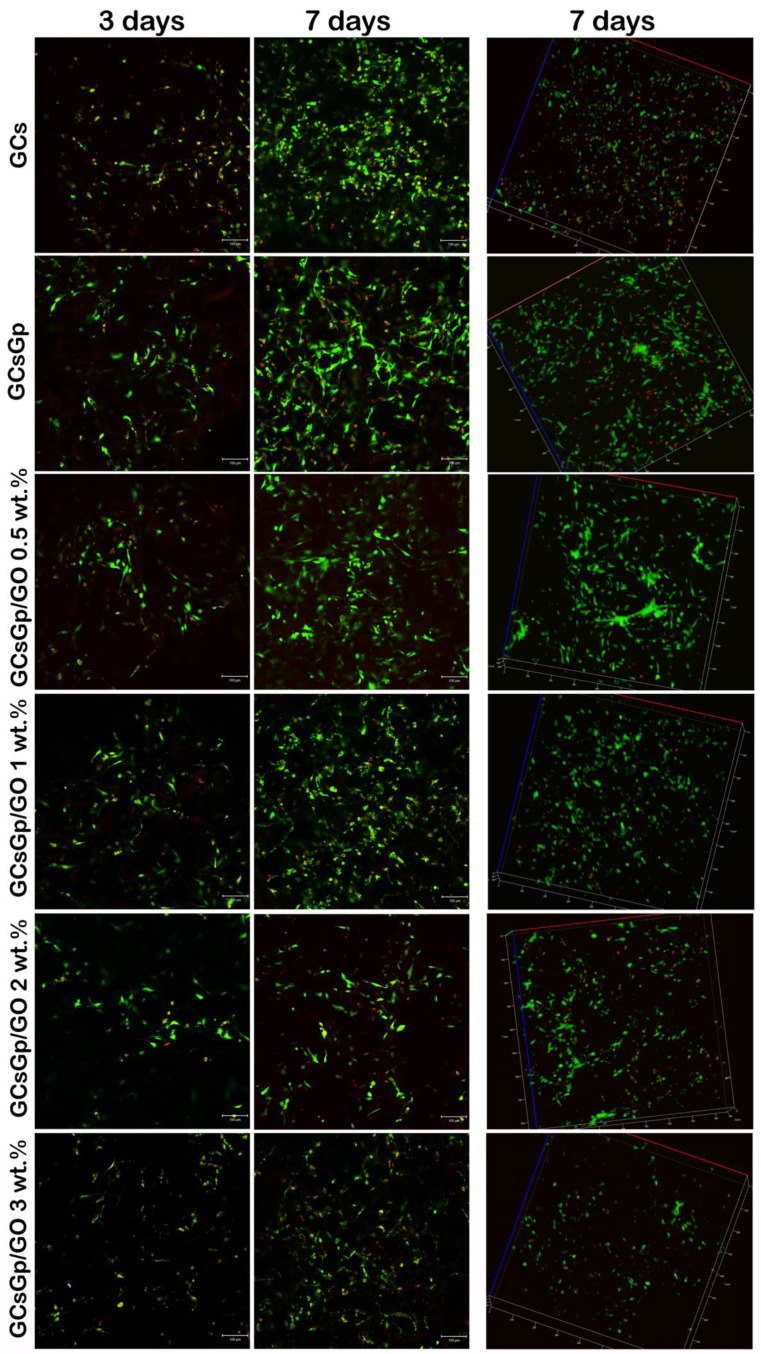
Fluorescence microscopy evaluation of living (green-labeled) and dead (red-labeled) cells in contact with GCs and GCsGp/GO scaffolds during one week of in vitro cell culture.

Qualitative evaluation of cell viability and proliferation in contact with GCsGp/GO scaffolds by Live/Dead assay (Figure 7) confirmed the results obtained by MTT and LDH assays, indicating that murine preosteoblasts proliferated and maintained cell viability on almost all tested composites from 3 to 7 days of culture. 3 days post seeding, all scaffolds present approximately the same distribution and number of cells. However, on GCs and GCsGp controls, more red-labeled cell nuclei than on the other composites could be highlighted. On the other hand, when the number of dead cells is normalized to the total number of cells in culture, then the general cytotoxicity level resembles the profile found by LDH assay.

The fluorescent images have also shown that the amount of green-labeled cells decreases once the concentration of GO rises after 7 days of culture. The proliferation occurred on all materials except GCsGp/GO 3 wt.%, where no major change can be observed from 3 to 7 days of culture. Thus, increasing GO concentrations over a particular threshold can restrict cell proliferation. In conclusion, the lowest cytotoxicity levels correlated with high cell viability were found for GCsGp/GO 0.5 wt.%.

### 3.7. In Vivo Results

**Clinical observation of the experimental animals**

The mice showed no postsurgical complications. Furthermore, no animals died post implantation and no inflammation or infections in the injured areas were detected.

**In vivo biocompatibility of GCSGp/GO scaffolds**

**Figure 8:**
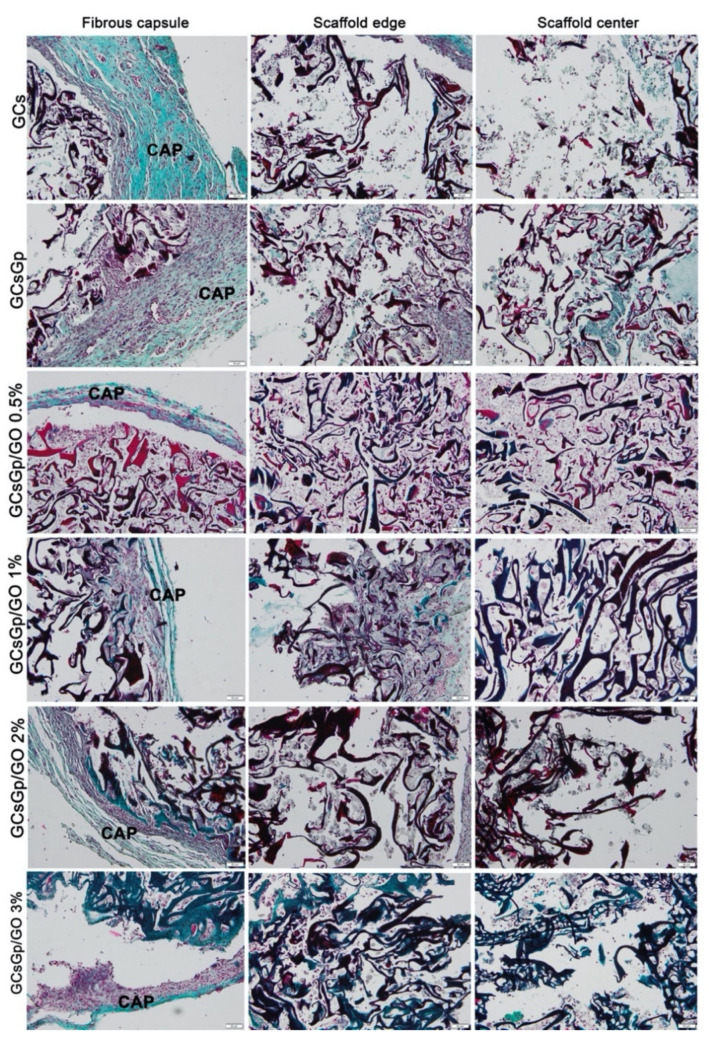
Light images of Gomori-trichrome-stained scaffolds at week 4 post implantation of GCs, GCsGp, GCsGp/GO 0.5%, GCsGp/GO 1%, GCsGp/GO 2%, and GCsGp/GO 3%, respectively, showing the varying thickness of the capsules, “CAP”, surrounding the different scaffolds (first column), and the histological aspect of the edge and center of scaffolds (second and third column, respectively). Collagen = green; scale bar = 50 μm.

As indicated by Gomori’s trichrome staining in green, thicker collagen deposition was found surrounding the GCsGp/GO scaffolds, as compared to GCs and GCsGp ones (Figure 8).

Cell infiltration into GCsGp/GO scaffolds occurred from the edge to the center, and the maximum deposition of the matrix was registered for GCsGp/GO 0.5% (Figure 8).

Degradation of scaffolds was observed as a loss of the regular network appearance and its replacement by cells and matrix. Remnants of scaffold material could be observed mainly at the center, showing that the scaffolds degraded more at the periphery than at the core.

**Figure 9:**
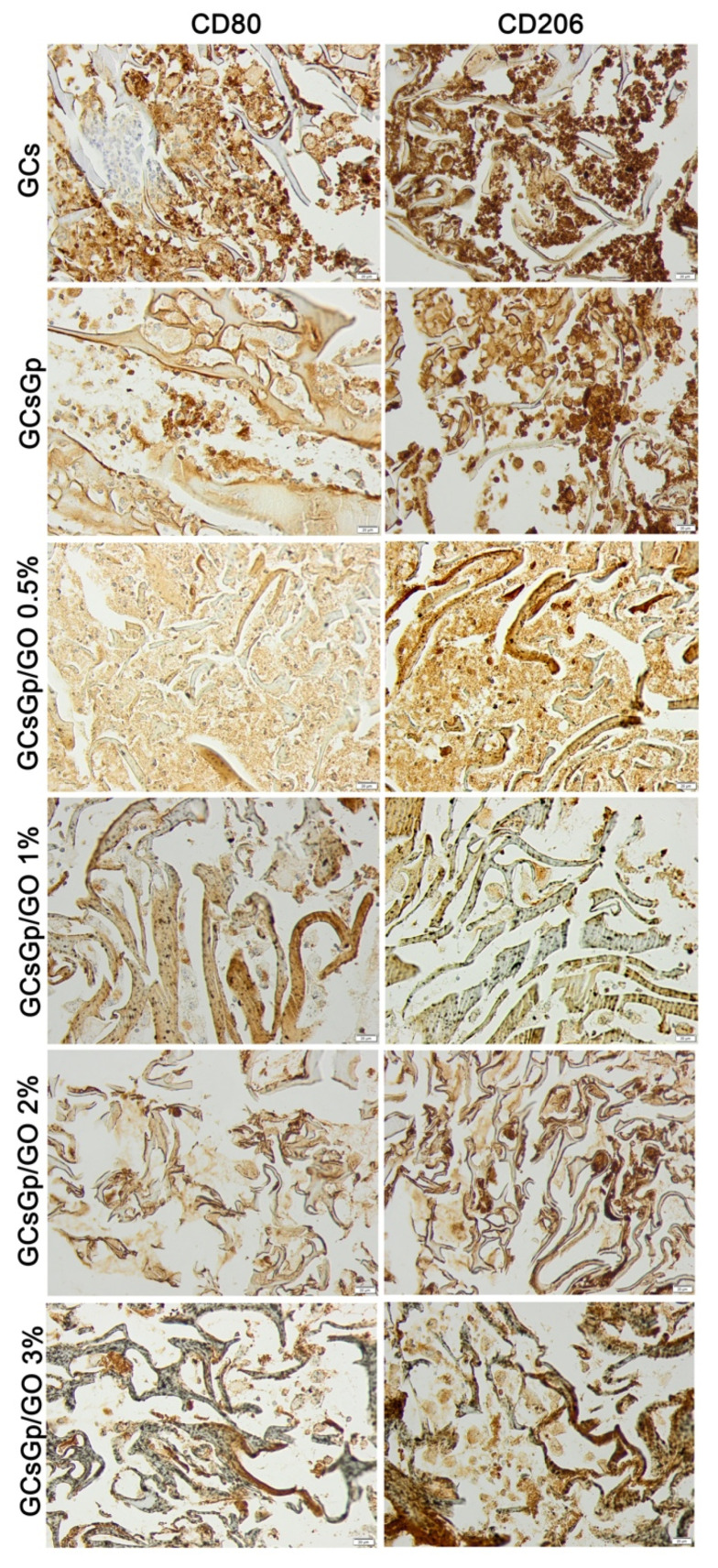
Immunohistochemical expression of CD80 and CD206 at week 4 post implantation for GCs, GCsGp, GCsGp/GO 0.5%, GCsGp/GO 1%, GCsGp/GO 2%, and GCsGp/GO 3%.

Foreign body giant cells (FBGC) were not seen at week 4 in any of the groups. Immunohistochemical analysis of CD80 (M1 marker) and CD206 (M2 marker) showed the highest expression for GCS and decreased for the GCsGp/GO batch, with a minimum reaction for GCsGp/GO 0.5% (Figure 9). In general, the overall decrease of the inflammatory profile could be ranked as: GCs > GCsGp > GCsGp/GO 1% ~ GCsGp/GO 2% ~ GCsGp/GO 3% > GCsGp/GO 0.5%.

## 4. Discussion

The specific extent of water uptake of the biocomposites is one of the pivotal features that prescribe the in vivo course of the implants. The swelling degree was most constant in the case of GCsGp, whose profile remained almost at the same level after 1 h. On the other hand, we noticed fast and dynamic swelling kinetics in the case of GCs, favored by unconstricted chains with total degrees of freedom. Amongst the controls, GCsGp showed the lowest rehydration caliber (SD value of 2000%—Figure 1), since genipin crosslinking induces polymer network stiffening and a certain degree of chain alignment. In addition to crosslinking, the compositing layout strongly impacts the mechanism of water assimilation as resulted from the mass gain of the specimens. Upon GO addition, it was expected that the bicomponent framework with hydrophobic domains would decrease the composite’s affinity to water. By increasing the amount of GO, the association between polymers and the carbon sheets leads to fluctuating rehydrating potential. The lowest amounts of GO (GCsGp/GO 0.5 and GCsGp/GO 1) affected the hydrophobicity the most due to better dispersibility and minor reaggregation of the GO sheets. Comparable values of 1470% and 1320%, respectively, were ascertained for the equilibrium SD of the two compositions. The highest degree of rehydration was logged in the case of the 2% supplemented composite (SD = 2050%), comparable to the crosslinked reference. Further addition of GO in GCsGp, nonetheless, decreases water retention (SD = 1560%).

This may be due to more hydrophobic slots exposed from within the surfaces of the highly porous dry network. Even though the decrease is not linear and inversely proportional with GO content, the swelling degree could be favored by the cloaking of GO sheet stacks at superior concentration and slightly influenced for the 3% GO ratio by crossing a threshold of polymer enveloping capacity.

A peculiar phenomenon was noticed in the case of GCsGp/GO 3 composition. The maximum SD was reached 2 h after immersion in PBS; henceforth, the network expelled part of the retained water, describing the so-called overshooting phenomena [20], albeit to a minor extent. The gradual deswelling is a consequence of the polymer network densification, owing to a dynamic process of hydrogen-bond formation between unreacted carboxyl groups of the polymer blend and GO veiled sheets when swollen. This oscillation can be attributed to a synergistic effect of macromolecular relaxation in the network and a thorough penetration of the PBS solution within the gel framework before achieving the final equilibrium state.

Distinct water-retaining outlines can be due to inherent relations between scaffold anisotropy and the swelling of this composite. In general, prediction and theorization of penetrant-induced water uptake is based on free energy considerations. So, by mixing the polymer-based composite with the penetrant, enthalpy and entropy changes contribute to the free energy mediating the swelling extent and to elastic deformations of the systems in conjunction with the stiffness induced by GO additivation [21].

Physicochemical properties, such as composition and pore template, play a major role in the unfolding of biodegradation within a given bioactive environment. Among the materials hereby investigated, GCsGp/GO 3 sponge exhibited the highest resistance to enzyme degradation while the uncrosslinked control degraded at the fastest rate. The GCsGp/GO 2 and GCsGp/GO 1 complete the top of the most COLase II retardant formulations. With respect to GCs, the high susceptibility to COLase II infers from two reasons related to structure and morphology: i. at molecular level, the polymer chains are loosely packed and less dense, since the network is not chemically crosslinked, and ii. at microscale level, whereby the total porosity and specific surface are the highest (Table 1) enabling a thorough penetration of the bioaggresive solution. Structure thickness, a measure of the average wall thickness, was also both the tiniest and the narrowest (19.2 ± 0.4 µm) in the control case. The unconstricted structuration of GCs is one of the reasons it features the highest SD. The packing density of the materials increased by Gp crosslinking mirrors in the total porosity (5% ⭨), wall size (25% ⭧), and specific surface (20% ⭨). Originating in this conjuncture, we hypothesize that inhibition of COLase II is due to attenuated infiltration within the specimens’ bulk and its interference with denser terrains and less interfacial surfaces. In addition, the GO rich composites swell dramatically less than the references due to the rigidity induced within the walls by GO embedding. Congruously, GO can endow the enveloping matrix with superior stability in aqueous media by supplying carboxyl groups essential to ensuring additional scaffolding with newly formed H bonds.

Referring to the overall trend, for Gp crosslinked samples, a pregnant weight loss flare occurs shortly after the beginning of the degradation treatment (1 h). For the next intervals, up to 12–24 h, the weight loss values describe a rather linear plateau, as weight differences oscillate between 15–20% in Δ*t*1 = *t*24 h − *t*1 h. Variations from 24 h to 90 days depict an escalating trend associated with 10–28% weight loss (Δ*t*2 = *t*90 d − *t*24 h). Small differences between Δ*t*1 (23 h) and Δ*t*2 (89 days) testify that enzymatic attack occurs with more potency at the beginning of the treatment. In time, the crosslinked specimens exhibit prolonged resistance against degradative media. In addition, the GO-loaded samples are less prone to degradation (4–10% more stable than GCsGp). This GO effect we monitored is in opposition to Saravanah et al. who reported more pronounced degradation in the case of gelatin–chitosan GO composites. Nonetheless, their networks were not crosslinked, providing the circumstances for the structures to become more brittle and less consolidated after GO saturation [13]. In our case, GCs’ reference catered the poorest stability against degradation due to the unassembled polymer networks.

From a morphological point of view, inner architecture of the biopolymer-GO composite scaffolds is fairly akin. GCs and GCsGp (Figure 3A–B) display comparable morphological features, with irregular pore sizes and shapes. Nonetheless, upon crosslinking, the pore walls become less slender and transparent. This is an evidence of the GCs framework thickening, which coheres with the confining effect Gp has towards polysaccharide and protein chains [22,23]. Since the crosslinked polymer chains possessed limited mobility, the pore formation dynamics was attuned accordingly.

Structural pore patterning is a measure of composite blocks dynamics and kinetics at molecular level, but it is also directly accountable for the quotas of total porosity (T.Po), structure thickness (St.Th), and specific surface (Sp.S) (reported in Table 1). T.Po assessed from µCT datasets is the volumetric ratio of all the pores in a given specimen. The values of T.Po are similar for all specimens, within the range of 86–90% of the total volume of a scanned object. On the other hand, important variations in St.Th appear. Gp generates a 25% increase in wall thickness compared to GCs reference, while GO increases with 5–30%. Sp.S influence is inversely proportional. The highest values for specific surface appear in the case of GCS and GCsGp/GO 0.5, which endorse them as highly promising for in vitro cell and in vivo tests. GCsGp/GO 2 and GCsGp/GO 3 could provide enhanced support due to more consolidated lateral sides, but their Sp.S is slightly lower, which could impair the kinetics of the multicomponent (cells and bioactive agents) native/simulated internal fluid.

Qualitatively, domains of comparable pore size are discernable for the GO-loaded formulations (Figure 3C2–F2). In addition, aligned assemblies of parallel pore arrays emerge with GO reinforcing. Interestingly, depending on the GO amount, a maximum degree of pore structuring stands out at 1 wt.% GO (Figure 3D1–D2). The incipient phase of regularized porosity is observed at 0.5 wt.% GO addition (Figure 3C1–C2). At higher concentrations, while pore morphogenesis perpetuates, size distribution widens and the even pattern moderately disarrays. By governing the GCs pore network, GO-loading could upgrade the biophysical properties of the polymer blend in terms of morpho-functional characteristics that promote cell affinity.

Moving on to addressing the biological activity of the composites, the performed biocompatibility assays (MTT, LDH, and Live/Dead) in this particular experimental set up demonstrated that GCs and GCsGp/GO composites are compatible with murine preosteoblasts. As stated in the literature [17], cell viability could be attenuated when cultured in GO-containing materials. The results indicated that increasing the content of GO over 3 wt.% did not have a beneficial effect on cells’ behavior. Even though cell proliferation was not promoted in higher GO load formulations, LDH assay revealed that during one week of culture, both controls, GCs, and GCsGp exhibited significantly higher cytotoxicity levels than GO-enriched materials. Therefore, it could be stated that higher GO amounts influenced cell response by inhibiting cell proliferation. Our results showed that the most optimal response of 3T3-E1 cells was found in contact with GCsGp/GO 0.5 wt.% in terms of viability and proliferation, thus proving that, for adequate cell responses, GO presence is required but in lower amounts. Our results are in accordance with other studies where the biocompatibility of GO with murine preosteoblasts was assessed. In this respect, Mehrali et al. demonstrated a positive interaction between preosteoblasts from the human fetal osteoblastic (hFOB) cell line and reduced GO up to a concentration of 1.5 wt.%, showing that the proprieties that GO brings to a scaffold have an important influence on cell behavior [24]. Moreover, Saravanan et al. generated a scaffold containing chitosan, gelatin, and GO, which was tested for its in vitro cytocompatibility with rat osteoprogenitors, concluding that the presence of 0.25% GO in the scaffolds’ structure delivered a better cell viability than the polymer control [25]. In conclusion, the most biocompatible composite in our study turned out to be GCsGp/GO 0.5 wt.% and could be validated for further applications in the field of BTE and regenerative medicine.

It is well known that chemical and mechanical properties of the material can affect biological response and modulate foreign body reaction. Biomaterials may induce significantly acute/chronic inflammatory responses, which can affect or delay tissue healing [26]. In contrast, the histopathological analysis showed that the subcutaneous implantation of the GCsGp/GO scaffolds to mice produced a minimum foreign body reaction, as evidenced by the presence of a thin fibrous capsule around the biomaterials and the lack of FBGC. This pathway is in the range of normal standard resolution process for biomaterials, mediated by a foreign body reaction [26].

It was likely that the degradation products of the GCsGp/GO scaffolds did not cause irritation of the surrounding tissue, allowing the tissue organization in the implantation area.

Incipient tissue formation at the periphery and the progression to the center in exchange with the degradation of the scaffold network was more balanced and advanced for GCsGp/GO 0.5% scaffold. We explain these results for GCS scaffolds versus those with GO through a biopolymer blend-genipin-GO synergistic effect. Regarding the difference between GCsGp/GO 0.5% and GCsGp/GO 1%, 2%, and 3%—probably the higher concentrations of GO on this new composition— encouraged an extended inflammatory response in the early post-implantation phase and degraded the scaffold network before cell migration and adhesion, consequently, unbalancing the healing process. These results and hypothesis also support the in vitro biocompatibility results, which showed GCsGp/GO 0.5%, ensuring the most optimal cellular response and lowest cytotoxicity. Moreover, Tenorio et al. (2019) determined that the biocompatibility of a graphene oxide and chitosan biocomposite—but without gelatin—implanted in Wistar rats’ critical size cranial bone defects for three months, finding less foreign body reaction for GO 0.5% than for GO 1%.

Degradable scaffolds, such as those tested here, are supposed to be phagocytized, a function attributed to M1 macrophages, followed by M2 phenotype activation, which attenuate acute/chronic inflammation and promote healing [27]. At week 4, we found an intense CD80 and CD206 immunopositivity for GCs and GCsGp/GO-subcutaneous implanted biomaterials, then for gelatin-chitosan–genipin-reinforced with graphene oxide, which suggested that the inflammatory process is still very active in the graphene-free scaffolds. The most balanced immune reaction was obtained for GCsGp/GO 0.5%, correlated with the more advanced tissue formation for this scaffold.

## 5. Conclusions

In this research, porous scaffold consisting of fish gelatin, chitosan, and GO were synthesized and characterized in the endeavor to address a shortage of feasible BTE materials. GCs scaffolds feature appealing properties that are regulated by GO proportion, whose fine-tuning properties were encountered both in dry and wet states of the composites. Interestingly, the composite GCsGp with 0.5% GO turned out to exhibit a biofriendly environment for murine preosteoblast in terms of in vitro viability and proliferation. Therefore, the scaffold offered an optimal morphological structure that allowed cells to adhere and grow significantly. The hypothesis that GO incorporation would generate distinct levels of cytotoxicity was confirmed. According to in vitro assays, low and moderate (0.5 and 1 wt.%) amounts of GO endow superior cytocompatibility and viability to the polymer control. Similar, the in vivo investigations revealed a minimum foreign body reaction, allowing for the host’s tissue to reorganize in the implantation area. Of all tested composites, GCsGp with 0.5% GO presented a significant incipient tissue formation at the periphery and a controlled immune response. These findings support the validation of a GCsGp-reinforced GO composite for future BTE applications. A better understanding of the patterning mechanisms of GO networks could valuably contribute to the generation of tunable formulations for a rich array of applications in biomedical engineering.

## Figures and Tables

**Table 1:** Summary of the percentual T.Po, metric St.Th and Sp.S of the 6 specimens investigated by means of µCT.

Sample	T.Po [%]	St.Th. [µ]	Sp.S [µ-1]
GCs	90.9 ± 1.6	19.2 ± 0.4	0.17 ± 0.002
GCsGp	87.5 ± 1.6	25.3 ± 1.5	0.13 ± 0.005
GCsGp/GO 0.5 wt.%	89.9 ± 0.6	20.0 ± 0.2	0.16 ± 0.002
GCsGp/GO 1 wt.%	86.7 ± 0.2	27.4 ± 0.6	0.13 ± 0.004
GCsGp/GO 2 wt.%	89.3 ± 1.8	23.5 ± 0.3	0.14 ± 0.002
GCsGp/GO 3 wt.%	89.96 ± 0.8	23.2 ± 0.1	0.14 ± 0.002
