# Correction: Vlasceanu et al. Comprehensive Appraisal of Graphene–Oxide Ratio in Porous Biopolymer Hybrids Targeting Bone-Tissue Regeneration. *Nanomaterials* 2020, *10*, 1444

**DOI:** 10.3390/nano15151207

**Published:** 2025-08-07

**Authors:** George Mihail Vlasceanu, Aida Șelaru, Sorina Dinescu, Cornel Balta, Hildegard Herman, Sami Gharbia, Anca Hermenean, Mariana Ionita, Marieta Costache

**Affiliations:** 1 Faculty of Medical Engineering, University Politehnica of Bucharest, Gh. Polizu 1-7, 011061 Bucharest, Romania; vlasceanu.georgemihail@yahoo.ro; 2 Advanced Polymer Materials Group, University Politehnica of Bucharest, Gh. Polizu 1-7, 011061 Bucharest, Romania; 3 Department of Biochemistry and Molecular Biology, University of Bucharest, Spl. Independentei 91-95, 050095 Bucharest, Romania; aida.selaru@bio.unibuc.ro (A.Ș.); sorina.dinescu@bio.unibuc.ro (S.D.); samithgh2@hotmail.com (S.G.); anca.hermenean@gmail.com (A.H.); marietacostache@gmail.com (M.C.); 4 Department of Immunology, National Institute for Research and Development in Biomedical Pathology and Biomedical Sciences “Victor Babes”, Spl. Independentei 99-101, 050096 Bucharest, Romania; 5 Research Institute of the University of Bucharest, 050095 Bucharest, Romania; 6 Aurel Ardelean Institute of Life Sciences, Vasile Godis Western University of Arad, Rebreanu 86, 310414 Arad, Romania; baltacornel@gmail.com (C.B.); hildegard.i.herman@gmail.com (H.H.)

In the original publication [1], a mistake was made in some of the subfigures in Figure 7. Misplacement of two images occurred during the processing of the figure (in the row of 3D representations at 7 days, more specifically between two samples, GCsGp/GO 1 wt.% and GCsGp/GO 2 wt.%) where the 3D representations were unintentionally switched between these two samples. The corrected Figure 7 appears below.

The authors state that the scientific conclusions are unaffected. This correction was approved by the Academic Editor. The original publication has also been updated.

## Figures and Tables

**Figure 7:**
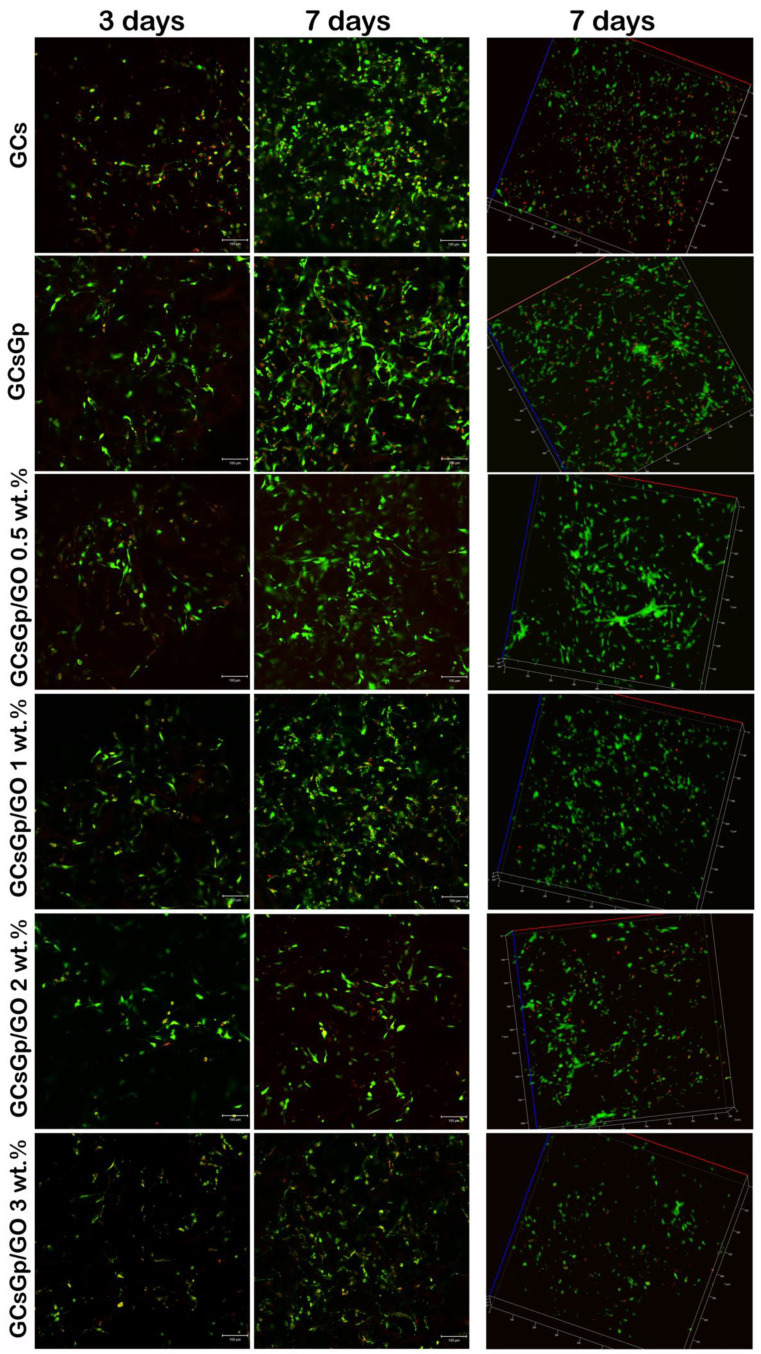
Fluorescence microscopy evaluation of living (green-labeled) and dead (red-labeled) cells in contact with GCs and GCsGp/GO scaffolds during one week of in vitro cell culture.
